# Seasonal Differences in Fecal Microbial Community Structure and Metabolism of House-Feeding Chinese Merino Fine-Wool Sheep

**DOI:** 10.3389/fvets.2022.875729

**Published:** 2022-03-23

**Authors:** Xingxing Zhang, Chuang Li, Khuram Shahzad, Mengli Han, Yanhua Guo, Xin Huang, Tongzhong Wu, Limin Wang, Yiyuan Zhang, Hong Tang, Qian Zhang, Mengzhi Wang, Ping Zhou, Fagang Zhong

**Affiliations:** ^1^State Key Laboratory for Sheep Genetic Improvement and Healthy Production, Xinjiang Academy of Agricultural and Reclamation Science, Shihezi, China; ^2^Institute of Animal Husbandry and Veterinary, Xinjiang Academy of Agricultural and Reclamation Science, Shihezi, China; ^3^College of Animal Science and Technology, Yangzhou University, Yangzhou, China; ^4^Department of Biosciences, COMSATS University Islamabad, Islamabad, Pakistan

**Keywords:** microbial community structure, season, Chinese merino fine-wool sheep, 16S rRNA gene, metabolic pathway, blood routine

## Abstract

The digestive tract microorganisms play a very important role in the host's nutrient intake, environmental suitability, and affect the host's physiological mechanism. Previous studies showed that in different seasons, mammalian gut microbes would be different. However, most of them are concentrated in wild animals. It remains unclear how seasonal change affects the gut microbes of Chinese merino fine-wool Sheep. Therefore, in this experiment, we continuously collected blood and feces samples of 50 Chinese merino fine-wool sheep in different seasons, measured the physiological indicators of blood, and passed 16S rRNA amplicon sequencing, determined the microbial community structure of fecal microorganisms and predicted flora function by PICRUSt. The results of blood physiological indicators showed that WBC, Neu and Bas in spring were significantly higher than those of other seasons. Fecal microbial sequencing revealed seasonal changes in gut microbial diversity and richness. Among them, Chinese merino fine-wool sheep had the highest gut microbes in summer. Firmicutes and Bacteroidetes were the dominant phyla, and they were unaffected by seasonal fluctuations. LEfSE analysis was used to analyze representative microorganisms in different seasons. The Lachnospiraceae and its genera (*Lachnospiraceae_NK4A136_group, Lachnospiraceae_AC2044_group, g_unclassified_f_ Lachnospiraceae*) were representative microorganisms in the three seasons of spring, summer and winter with harsh environmental conditions; while in autumn with better environmental conditions, the Ruminococcaceae and its genus (*Ruminococcaceae_UCG-009* and *Ruminococcaceae_UCG-005*) were the representative microorganism. In autumn, the ABC transporter and the pyruvate metabolic pathway were significantly higher than other seasons. Correlation analysis results showed that Lachnospiraceae participated in the ABC transporters metabolic pathway, which caused changes in the blood physiological indicators. Overall, our results showed that, in response to seasonal changes, Chinese merino fine-wool sheep under house-feeding have adjusted their own gut microbial community structure, causing changes in the metabolism, and thus changing the physiological conditions of the blood. In the cold season, producers should focus on regulating the nutritional level of feed, enhancing the level of butyric acid in young animals to increase the ABC transporter, resist the external harsh environment, and improve the survival rate.

## Introduction

The gut microflora is complex, dynamic, and susceptible to disturbances such as diet changes, environmental factors, and intestinal pathogens (1, 2). These are crucial for the host's dietary intake, metabolic behavior, immunological response, development, and growth (3, 4). Seasonal climate transitions have been reported to cause significant changes in the composition of mammalian microbiota (1, 5, 6). It was also shown by longitudinal analysis of animal gut microbiota, such as Hadza Hunter-Gatherers, Tamiasciurus hudsonicus and Ailuropoda melanoleuc (1, 5, 6). Most of the research on the dynamics of mammalian gut microbiota was conducted in stress-free environments, and there was no quantitative dietary information available related to habitat. However, recent reports on the composition of the gut microbiota of herbivores located in the semi-arid savannah of East Africa showed that the seasonal turnover and microbiota association of domestic species was greater than that of wild species (7). These findings suggested that seasonal changes were associated with host gut microbes. Despite this, we are not aware of any studies on the association between seasonal variations and gut microbiota in domestic mammals such as sheep.

Climate change can directly affect animals and alter physiological indicators (8). Physiological functions are significantly influenced by changes in ambient temperature and humidity. The phenomenon that mammals are sensitive to heat stress, especially small ruminants, has been widely reviewed in the literature (9). In addition, cold stress is a huge challenge to the survival of lambs. In Xinjiang, the lamb survival rate is much lower than the annual average survival rate (10, 11). The fact that climate change cause huge losses to the production of animal husbandry requires detailed research to better understand the mechanisms by which animals adapt to climate related transitions (12).

Located in an important position along the Silk Road, Xinjiang is the core area of the current “One Belt, One Road” development plan, and is also one of the regions with the greatest potential for the development of agriculture and animal husbandry in China (13). Xinjiang is located in the hinterland of Eurasia, far away from water vapor sources, with sparse precipitation, dry climate or semi-arid regions (14). Chinese merino fine wool Sheep is the first fine-wool sheep in China announced by the Ministry of Agriculture in 1954, and is the largest number of Chinese merino fine-wool sheep breeds in China (15). It belongs to a temperate continental climate and is a typical feature of arid reclamation type. Ruminants are highly dependent on the gut microbiota to decompose and digest feed (16). However, the relationship between the blood physiological indicators of Chinese merino fine-wool sheep and the intestinal microbiota with seasonal changes in natural habitats is still unclear, despite of the information available. It may be helpful to understand sheep's adaptation to seasonal climate changes.

Thus, we hypothesized that the temperature and humidity might mediate the season and resultantly influence gut microbes, which in turn affect changes in blood physiological indicators and have a certain impact on animal health. We performed 16S rRNA gene analysis of intestinal flora and blood physiological indicator measurement on Chinese merino fine-wool sheep in four seasons in Shihezi, Xinjiang, to investigate the impact of seasonal changes on intestinal flora and blood physiological indicators. These in-depth and longitudinal analyses contribute to understanding seasonal physiological and microbial conditions with theoretical implications for improving lamb's survival rate.

## Materials and Methods

### Location and Experimental Animals

The experiment was carried out in Chinese merino fine-wool sheep farm in Ziniquan Town (85°76′19.75′E, 43°99′31.98′N), Shihezi City, Xinjiang. The climate in this area is temperate continental climate, with hot and dry summers and harsh environmental climates in winter. The temperature during summer and winter ranges from −29 to 37 °C, which is typical for arid or semi-arid regions. The annual average temperature is 10 ± 1.0°C, and the annual average air humidity is 77.6 ± 2.0%. Shihezi is the central city of the Wu–Chang–Shi urban group, and is located in the heart of “the One Belt, One Road.” During the heating season (October 15 to April 15 of the following year), Shihezi air pollution is serious, and the average PM_2.5_ concentration exceeds the national standard daily value of 75 μg / m^3^ (GB3095-2012) (17).

Before the start of the experiment, we randomly selected 50 Chinese merino fine-wool sheep from the farm and marked them. The sheep (average age 2.0 ± 1.0 years) were chosen according to the following criteria: no pregnancy throughout the trial period, clinically healthy, and no symptoms of disease after a thorough clinical examination (average rectal temperature 38.5°C, rumen movement 3-4 / 2 min). According to the NRC (2007) mutton sheep nutritional requirements standard, the test diet was prepared (Supplementary Table 1). The sheep were fed (9: 00 and 18: 00 h) twice per day. The farm was monitored for infectious diseases and routine deworming was carried out twice a year.

### Sample Collection

In our research, the sampling period spanned from 2018 to 2019. According to the climate in the sheep farm in previous years, the rectal stool and blood samples were collected at a time point with obvious seasonal characteristics. Two hundred stools and blood samples were collected. For the samples, the sampling times were December 29, 2018 (Winter), March 29, 2019 (Spring), July 3, 2019 (Summer), and October 1, 2019 (Autumn). We hung an automatic temperature and humidity recorder (Elitech GSP-8A) at a distance of 0.60 m from the ground in the sheep barn to measure the temperature and humidity in the barn. The recorder automatically records the temperature (TH) and humidity (RH) every 30 min, and calculates the average temperature and humidity on each collection day (Table 1). Temperature humidity index (THI) calculation formula is: THI = (1.8 × T+32) − [(0.55 – 0.0055 × RH) × (1.8 × T−26)] (18). We used sterile gloves to collect fecal samples, stored them in the DNase and RNase free tubes, immediately frozen in liquid nitrogen (−196°C) and then stored at −80°C. A blood sample was taken in each animal's carotid artery at the same time of which a fecal sample was collected. The vacuum tube for blood collection contained 10% EDTA anticoagulant. The blood sample was kept at 8°C and delivered to the lab for physiological index determination right away (19).

**Table 1:** Environmental index of the day at different sampling time points.

**Environment index**	**Winter**	**Spring**	**Summer**	**Autumn**
	**(2018.12.29)**	**(2019.3.29)**	**(2019.7.3)**	**(2019.10.1)**
High temperature^a^	−17°C	19°C	35°C	15°C
Low temperature^b^	−27°C	4°C	21°C	6°C
Relative humidity^c^	77.40	75.30	49.70	58.80
THI^d^	36.38	50.28	72.31	67.43
Air quality index^e^	115	51	70	24
PM${}_{2.5}^{\text{f}}$	86	22	22	13

a−c *High temperature, low temperature and relative humidity were measured values*.

d *THI, Temperature humidity index. THI was the calculated value*.

e, f *The air quality index and PM_2.5_ were from the local weather bureau*.

### Blood Routine Index Determination

The total number of blood samples were 200 (50 samples collected in each season). We measured neutrophils (Neu), basophils (Bas), haematocrit (HCT), the leukocytes (WBC), lymphocytes (Lymph), mean corpuscular hemoglobin concentration (MCHC), monocytes (Mon), eosinophils (Eos), percentage of neutrophils (Neu%), percentage of lymphocytes (Lymph%), percentage of monocytes (Mon%), percentage of eosinophils (Eos%), percentage of basophils (Bas%), erythrocytes (RBC), hemoglobin (HGB), mean corpuscular hemoglobin (MCH), coefficient of variation of red blood cell distribution width (RDW-CV), standard deviation of red blood cell distribution width (RDW-SD), platelet count (PLT), mean corpuscular volume (MCV), mean platelet volume (MPV), platelet distribution width (PDW), and platelet crit (PCT) in the blood using the five-differential blood cell analyzer BC-5000 Vet (Mindray, China).

### DNA Extraction and 16S rRNA Amplicon Pyrosequencing

Fecal DNA was extracted using fecal genomic DNA extraction kits (Solarbio, Beijing, China). A NanoDrop 2000 UV-Vis spectroscopy (Thermo Scientific, Wilmington, USA) was used to assess the quantity and purification of DNA. The DNA integrity was validated using a 1 percent agarose gel electrophoresis. The thermocycler PCR equipment was used to amplify the 16S V3 - V4 region using bacterial universal primers 338F (5′-ACTCCTACGGGAGGCAGCAG-3′) and 806R (5′-GGACTACHVGGGTWTCTAAT-3′) (GeneAmp 9700, ABI, United States).

20.0 μl total volume, 5 × Fast Pfu buffer 4 μl, 2 μl 2.5 mM dNTPs, primer 338F 5 μM, primer 806R 5 μM, Fast Pfu DNA 0.4 μl (TransGen, China), BSA 0.2 μl, template DNA 1.5 μl (10 ng), and dd H_2_O were added to 20 μl to make up the PCR reaction system. The PCR reactions we performed were as follows: 3 minutes of denaturation at 95°C, 27 cycles of 30 seconds at 95°C, 30 seconds for annealing at 55°C, and 45 seconds for elongation at 72°C, followed by a final extension at 72°C for 10 minutes. The PCR products were extracted from 2% agarose gels, then purified with AxyPrep DNA Gel Extraction Kit (TransGen, China) and performed with QuantiFluor^TM^-ST from QuantiFluor (Promega, USA). Finally, we collected the purified amplicons in equimolar solution and performed 2 ×300 bp analysis on the Illumina MiSeq platform (Illumina, San Diego, USA) according to Shanghai Majiebi Biopharmaceutical Technology Co., Ltd., China to the standard protocol.

### Sequence Data Processing

Fastq raw files were demultiplexed and filtered using QIIME (version 1.9.1). Operational taxonomic units (OTUs) were clustered using UPARSE (version 7.0.1090 http://drive5.com/uparse/) based on 97% similarity. Chimeric sequences were identified and removed by UCHIME (USEARCH, version 9.2). For the SILVA 16S rRNA database (SSU128, available May 2018), 16S rRNA gene sequences were classified and analyzed using the Bayesian algorithm of the RDP classifier (version 2.2) with 70% confidence limit.

### Statistical Analysis

Majorbio i-sanger cloud platform (Majorbio, China, http://www.i-sanger.com/) is a free online platform. It was used to analyze later data and outcomes. Sequence data analysis was mainly carried out using R (Version 3.3.1). Alpha diversity indexes (Shannon and Chao) were calculated by Mothur (Version v.1.30.2), and the difference between groups was tested by Kruskal rank-sum test. Dilution curve, venn diagram, Principal Co-ordinate Analysis (PCoA) and species stacking diagram are statistically analyzed and mapped by R (Version 3.3.1). The significance of microbiota structure differentiation in each group was evaluated by PERMANOVA (permutation multivariate analysis of variance) using R (Version 3.3.1). LEfSe analysis (http://huttenhower.sph.harvard.edu/galaxy/root?tool_id=lefse_upload) uses linear discriminant analysis (LDA > 3.5) on samples to identify communities or species that show substantial differences in sample division under various grouping criteria based on taxonomic composition. To begin, the software uses non-parametric factorial Kruskal-Wallis (KW) rank sum test to identify the characteristics and groups with significant abundance differences. Finally, LEfSe employs LDA to calculate the impact of each component's abundance on the differential effect. Phylogenetic investigation of communities by reconstruction of unobserved states (PICRUSt) was used to predict the metabolites of the microbial community and their enriched metabolic pathways, to perform function prediction. The predicted metabolic pathways used the non-parametric Wilcox test of SPSS 20.0 to analyze the differences. The variations between blood physiological markers in each group were investigated using SPSS (version 20.0) single-factor analysis of variance. The descriptive statistics of blood physiological indexes and KEGG signaling pathways were showed as mean ± standard deviation (mean ± SD). The mapping was done using R (version 3.3.1) to calculate the correlation and significance between different microorganisms and different metabolic pathways. *R* values > 0.5 or < −0.5 and *P*-values <0.05 were considered significant, where R represents the Spearman rank correlation coefficient (20).

## Results

### Microbial Diversity

**Figure 1:**
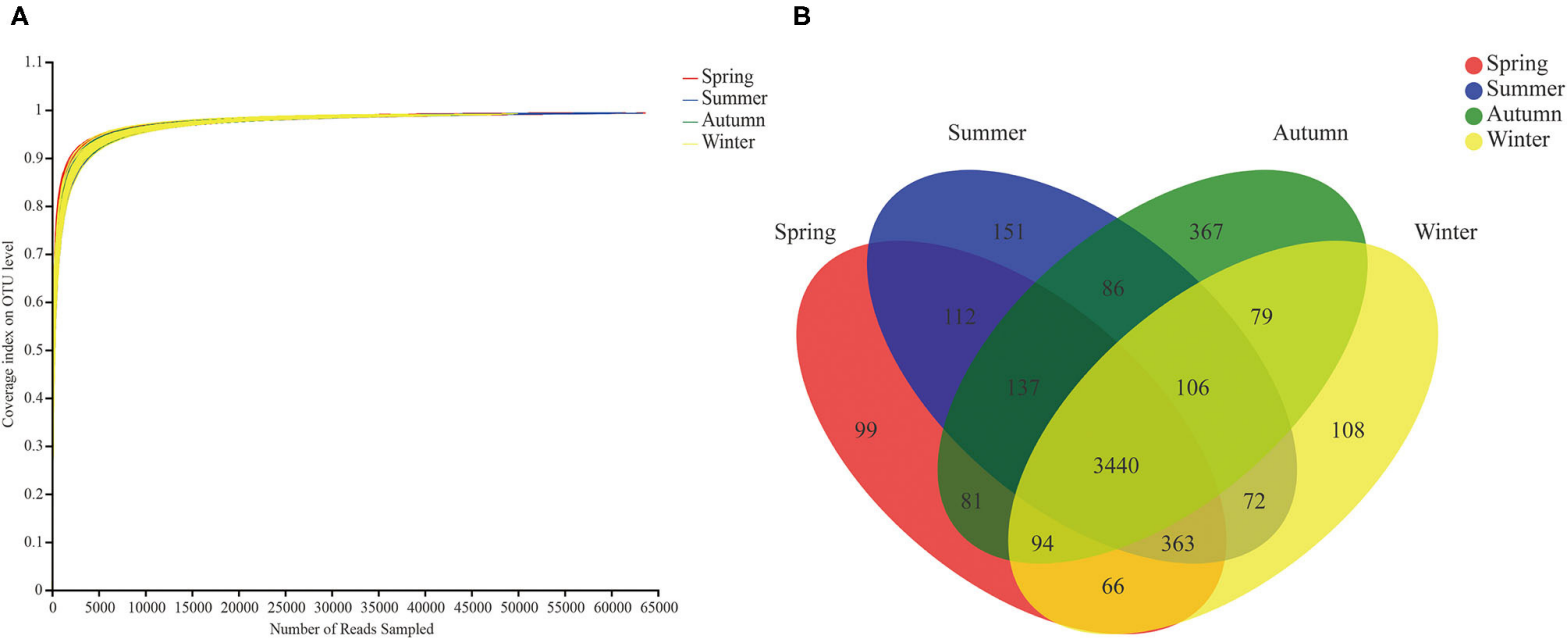
Rarefaction curves and Venn diagram. **(A)** Rarefaction curves of gut microbial communities. The rarefaction curve was drawn with the amount of extracted data as the abscissa and the coverage index value as the ordinate. **(B)** OTU Venn diagram in different seasons.

A number of 8,176,070 valid sequences were obtained after quality processing, with a minimum valid sequence of 19,573 and a maximum valid sequence of 63,704 (the average reads of 40,880). According to the rarefaction curve study, the sequencing data encompassed the majority of the microbial diversity in all samples (Figure 1A). These sequences were assigned to 5,361 OTUs with 97% similarity, of which 4 seasons shared 3,440 OTUs viz. Spring exclusive 99 OTUs, summer exclusive 151 OTUs, autumn exclusive 367 OTUs, and winter exclusive 108 OTUs (Figure 1B).

**Figure 2:**
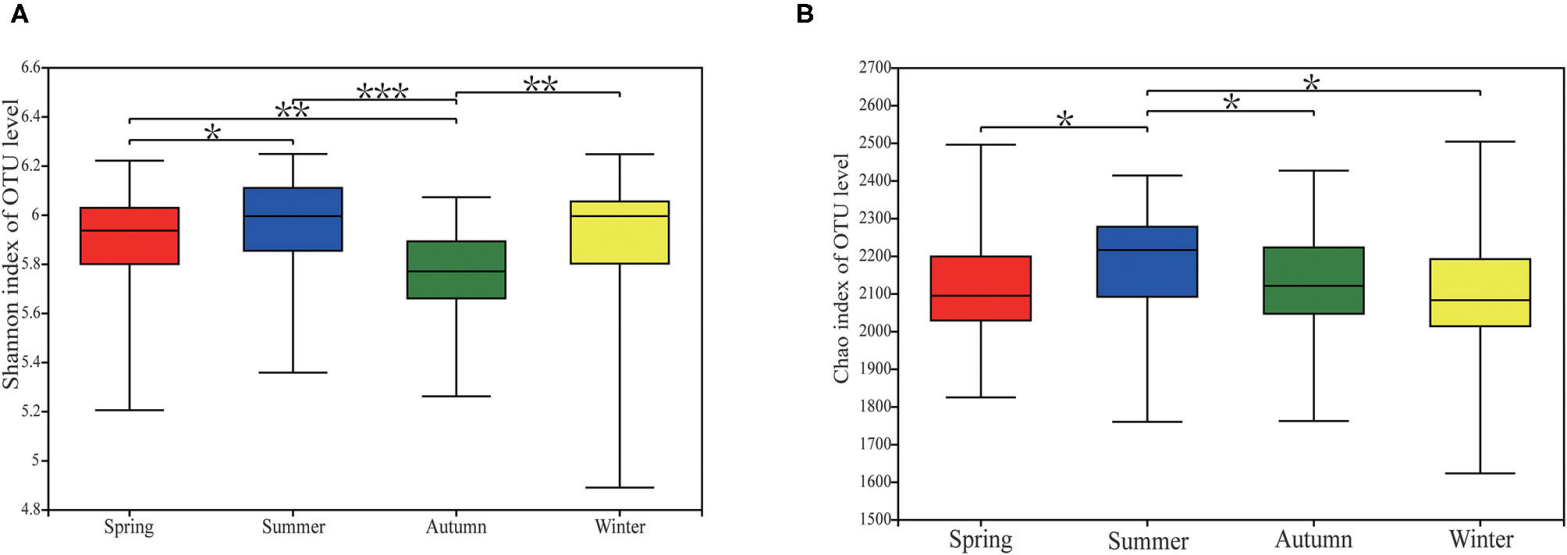
Alpha diversity of gut microbial community in Chinese merino fine-wool Sheep. **(A)** Shannon index. **(B)** Chao index. After the Kruskal-Wallis rank sum test, the differences in alpha differentiation between groups were statistically significant, as indicated by different letters (*P* ≤ 0.05). “*” means *P* ≤ 0.05, “**” means 0.001 < *P* ≤ 0.01, *** means *P* ≤ 0.001.

There were significant differences in alpha diversity of fecal microorganisms in different seasons. Its metrics (Shannon and Chao) showed the greatest diversity and richness of the fecal flora in summer (Figure 2). Shannon had significant differences between fall and spring, summer and winter (*P* < 0.05), and also between spring and summer (*P* < 0.05) (Figure 2A). The Chao index differed significantly between spring and summer (*P* < 0.05), summer and fall (*P* < 0.05), and summer and winter (*P* < 0.05) (Figure 2B).

### Cluster Analysis of Microbiota

**Figure 3:**
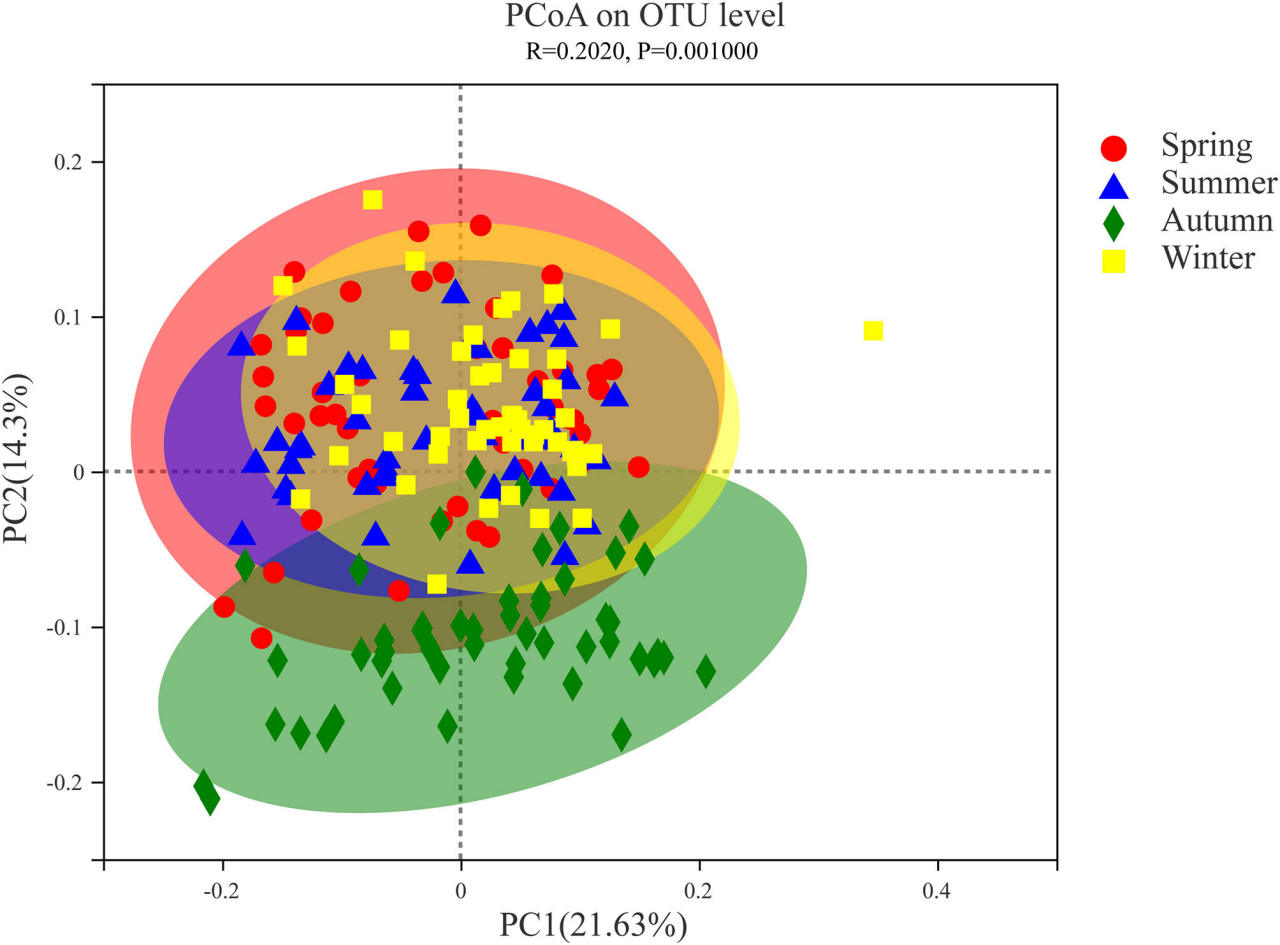
Principal coordinate analysis (PCoA) of fecal microflora of Chinese merino fine-wool Sheep in different seasons. PCoA was calculated from the weighted UniFrac metrics. Each point represents a single microbiota sample obtained from Spring (red circle), Summer (blue triangle), Autumn (green diamond), and Winter (yellow square) seasons.

PCoA based on weighted UniFrac metric data compares the microbiota of a single Chinese merino fine-wool sheep, and the results showed obvious clustering in different seasons (Figure 3). Among them, the spring, summer and winter samples clustered together, and only part of the flora clustered together with the other three seasons in autumn. The PERMANOVA results showed at least one significant difference (*P* ≤ 0.001) between any two groups (Supplementary Table 2). According to the sample distance matrix, the statistical comparison test was performed on the mean value of the sample distance in different seasons, the *t* test was performed on the samples in different periods, and the significance *P*-value of the statistical test was obtained by the Monte Carlo permutation test. Except for the fact that fecal microbial diversity of Chinese merino fine-wool sheep did not differ significantly between spring and summer (*P* = 0.115), it was significantly different between the any other two seasons (*P* ≤ 0.001) (Supplementary Table 3).

### Composition of Microbial Community Structure

**Figure 4:**
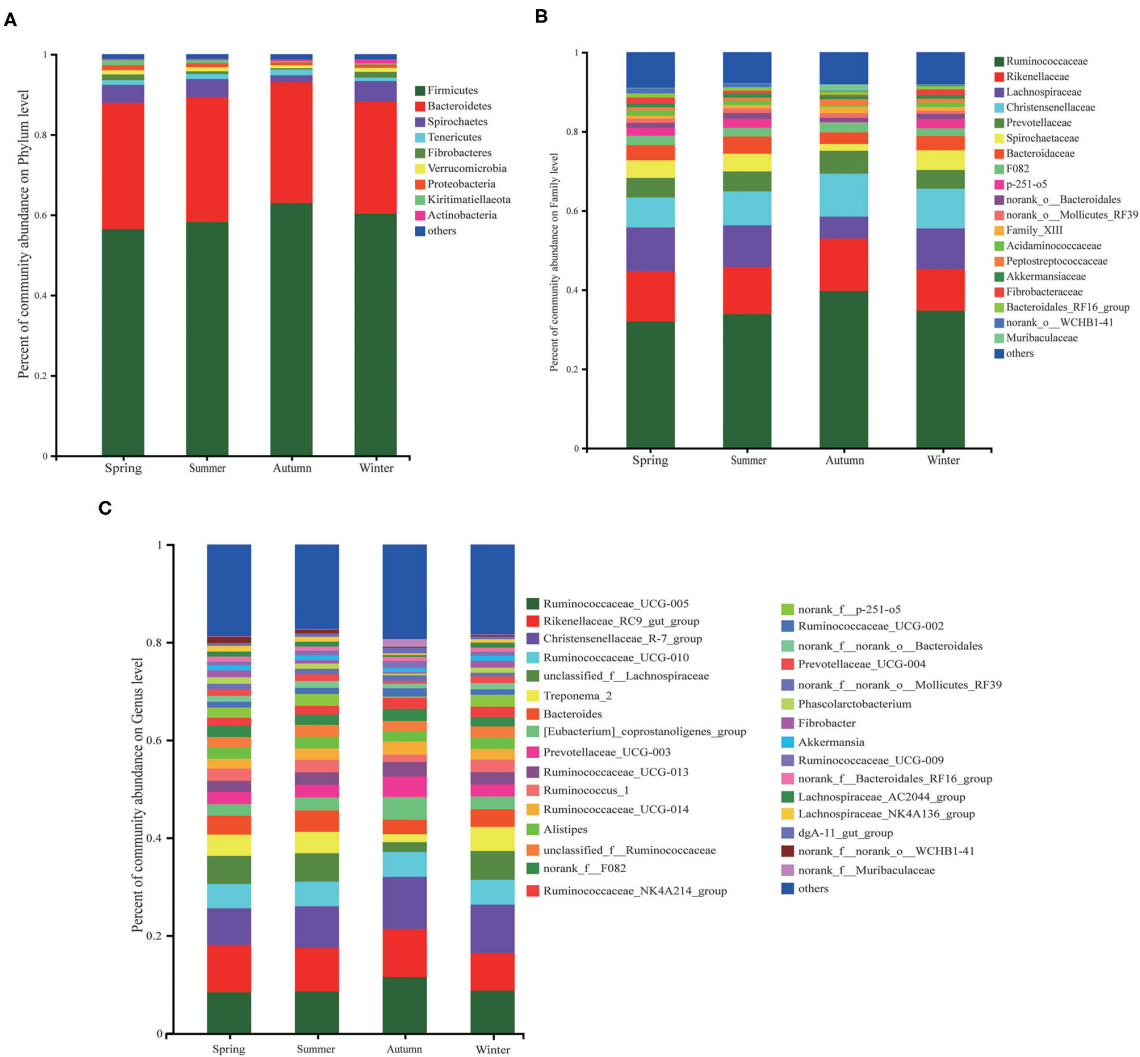
The relative abundance of microorganisms at the level of phyla, family and genus in different seasons (the abundance below 1% were merged with others). **(A)** The phyla level of fecal bacterial communities. **(B)** Fecal bacterial community at the family levels. **(C)** Fecal microbiota at the genus levels.

At the phyla level, Firmicutes and Bacteroidetes were dominated in the four seasons (Figure 3). Followed by the other 7 phyla, namely Spirochaetes, Tenericutes, Fibrobacteres, Verrucomicrobia, Proteobacteria, Kiritimatiellaeota, and Actinobacteria (Figure 4A). At the family level (Figure 4B), there were 19 families with a relative abundance of more than 1%. Ruminococcaceae and Rikenellaceae were the main families, followed by Lachnospiraceae, Christensenellaceae, Prevotellaceae, Spirochaetaceae, Bacteroidaceae, F082, p-251-o5, norank_o__Bacteroidales, norank_o__Mollicutes_RF39, Tablerep 3 and Family_XIII. At the genus level, *Ruminococcaceae_UCG-005, Rikenellaceae_RC9_gut_group*, and *Christensenellaceae_R-7_group* were the main bacterial genera, followed by *Ruminococcaceae_UCG-010, unclassified_f__Lachnospiraceae, Treponema_2, Bacteroides, [Eubacterium]_coprostanoligenes_group, Prevotellaceae_UCG-003* and *Ruminococcaceae_UCG-013* (Figure 4C).

### Comparison of Bacterial Flora

**Figure 5:**
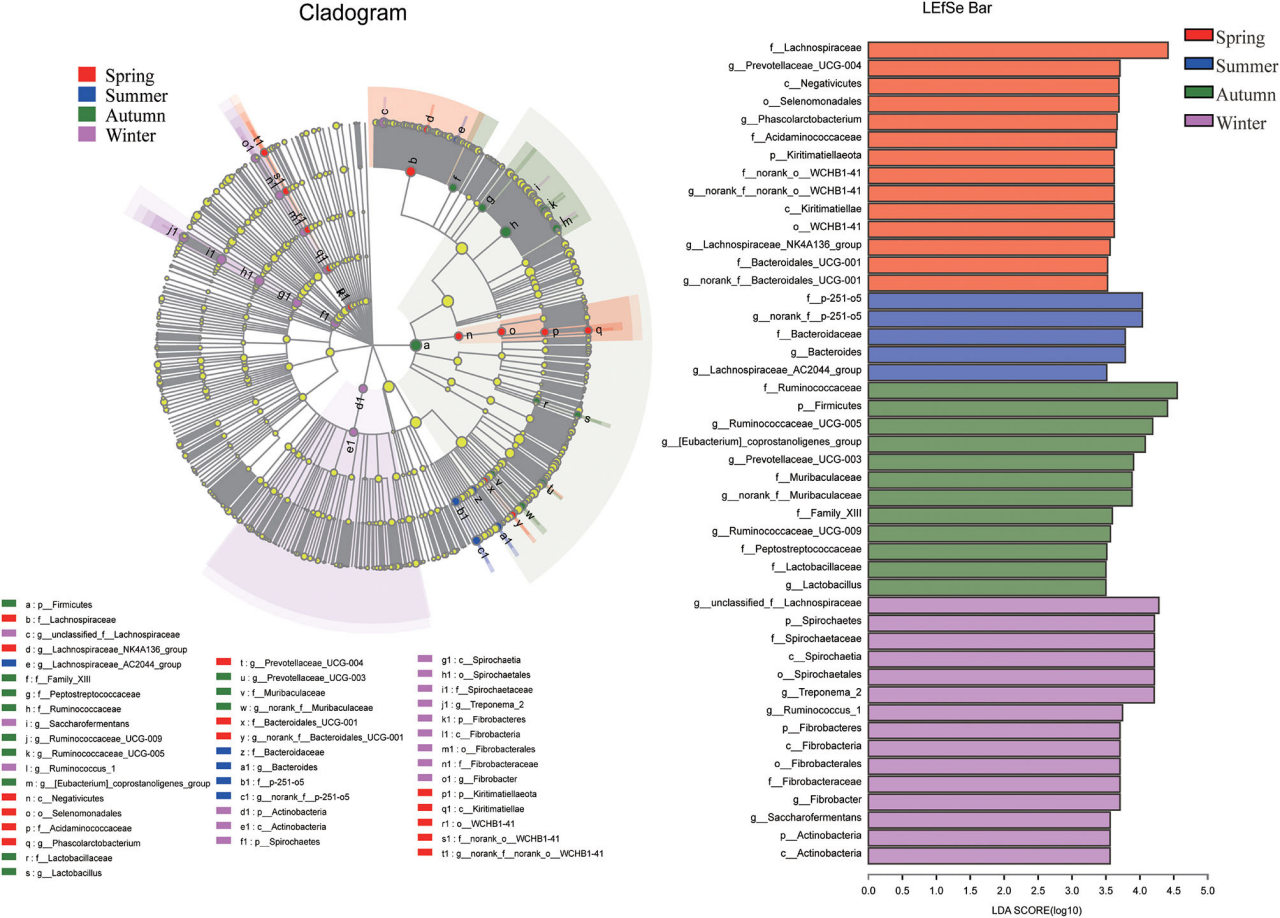
LEfSe analysis. There were statistically significant differences in abundance between any two seasons based on LDA distribution histograms and phylogenetic distribution of biomarkers.

In order to determine the abundance of different flora and the related categories of intestinal microbial community, LDA effect size (LEfSe) analysis (score > 3.5) was used to find biomarkers showing significant differences between groups (Figure 5). At phyla level, Kiritimatiellaeota had a higher relative abundance in spring. Firmicutes had a higher relative proportion in autumn. Moreover, the relative proportion of Spirochaetes, Fibrobacteres and Actinobacteria were higher in winter. At the family level, 14 representative microorganisms were obtained. The relative proportion of Bacteroidales_UCG-001, Lachnospiraceae, norank_o__WCHB1-41 and Acidaminococcaceae were relatively high in the spring season. In summer, relatively rich families were p-251-o5 and Bacteroidaceae. In autumn, Peptostreptococcaceae, Family_XIII, Ruminocobacaceae, Lacuritobaculaceae, and Lacuritobaculaceae were relatively enriched. Spirochaetaceae and Fibrobacteraceae were relatively abundant in the winter. At the genus level, LEfSE analysis was performed on 19 representative different bacterial genera. Among them, the relatively high abundance of bacteria in spring were *norank_f__norank_o__WCHB1-41, Phascolarctobacterium, Lachnospiraceae_NK4A136_group, Prevotellaceae_UCG-004*, and *norank_f__Bacteroidales_UCG-001*. The relative abundances of *Lachnospiraceae_AC2044_group, norank_f__p-251-o5* and *Bacteroides* were more abundant in summer. The genera with higher relative abundance in autumn were *Ruminococcaceae_UCG-009, Ruminococcaceae_UCG-005, [Eubacterium]_coprostanoligenes_group, Prevotellaceae_UCG-003* and *norank_f__Muribaculaceae*. The relative abundance of *g_unclassified_f_Lachnospiraceae, Fimabrobacter_2, Ruminococcaceae_UCG-003* and *norank_f__Muribaculaceae* were more abundant in the winter.

### Prediction of Intestinal Microbial Function by PICRUSt

We used PICRUSt to predict the function of the sequencing data. The analysis results of the third-level metabolic pathways of the KEGG pathway (Table 2) showed that the biosynthesis of secondary metabolites, microbial metabolism in diverse environments, ribosome, aminoacyl-tRNA biosynthesis, cysteine and methionine metabolism, pyruvate metabolism, oxidative phosphorylation, carbon fixation pathways in prokaryotes, glycine, serine and threonine metabolism in autumn were significantly induced than in any other seasons (*P* < 0.05). In addition, glycolysis, gluconeogenesis, nucleotide sugar metabolism, ABC transporters, purine metabolism, amino sugar and metabolic pathways in autumn were significantly lower than those in other seasons (*P* < 0.05). Biosynthesis of amino acids and quorum sensing in winter were significantly higher than those in other seasons (*P* < 0.05).

**Table 2:** PICRUSt shows the predicted relative abundance of all KEGG signaling pathways in fecal microbes (Level 3 Kos, > 1%).

**KEGG**	**Spring**	**Summer**	**Autumn**	**Winter**	***P*-value**
Metabolic pathways	18.30 ± 0.1a	18.27 ± 0.11ab	18.21 ± 0.12c	18.25 ± 0.09b	<0.001
Biosynthesis of secondary metabolites	9.19 ± 0.04b	9.18 ± 0.04c	9.21 ± 0.04a	9.18 ± 0.06c	0.001
Biosynthesis of amino acids	4.42 ± 0.06b	4.43 ± 0.06b	4.42 ± 0.08b	4.45 ± 0.11a	0.015
Microbial metabolism in diverse environments	4.32 ± 0.03b	4.33 ± 0.02b	4.34 ± 0.02a	4.33 ± 0.09b	<0.001
Carbon metabolism	2.86 ± 0.03b	2.87 ± 0.03b	2.91 ± 0.03a	2.86 ± 0.03b	<0.001
Ribosome	2.52 ± 0.04b	2.52 ± 0.03b	2.57 ± 0.03a	2.52 ± 0.08b	<0.001
ABC transporters	2.01 ± 0.11a	2.04 ± 0.14a	1.86 ± 0.13b	2.06 ± 0.12a	<0.001
Purine metabolism	1.59 ± 0.01a	1.59 ± 0.02a	1.57 ± 0.02b	1.58 ± 0.02a	<0.001
Two-component system	1.56 ± 0.06b	1.58 ± 0.06ab	1.58 ± 0.07a	1.59 ± 0.06a	0.028
Quorum sensing	1.35 ± 0.05b	1.36 ± 0.05b	1.35 ± 0.07b	1.38 ± 0.04a	0.035
Pyrimidine metabolism	1.26 ± 0.01	1.26 ± 0.01	1.25 ± 0.01	1.25 ± 0.02	0.166
Aminoacyl-tRNA biosynthesis	1.15 ± 0.02b	1.15 ± 0.02b	1.16 ± 0.02a	1.15 ± 0.03b	<0.001
Glycolysis / Gluconeogenesis	1.14 ± 0.01a	1.14 ± 0.01a	1.13 ± 0.01b	1.13 ± 0.01a	<0.001
Cysteine and methionine metabolism	1.10 ± 0.02c	1.10 ± 0.02c	1.12 ± 0.02a	1.10 ± 0.03b	<0.001
Amino sugar and nucleotide sugar metabolism	1.10 ± 0.02a	1.10 ± 0.02a	1.06 ± 0.02b	1.10 ± 0.02a	<0.001
Pyruvate metabolism	1.06 ± 0.02b	1.06 ± 0.02b	1.10 ± 0.02a	1.07 ± 0.02b	<0.001
Oxidative phosphorylation	1.07 ± 0.03b	1.07 ± 0.02b	1.08 ± 0.04a	1.06 ± 0.03b	0.001
Carbon fixation pathways in prokaryotes	1.01 ± 0.03b	1.01 ± 0.03b	1.04 ± 0.02a	1.00 ± 0.03b	<0.001
Glycine, serine and threonine metabolism	1.00 ± 0.01b	1.00 ± 0.01b	1.01 ± 0.01a	1.00 ± 0.01b	<0.001

*The value shown are the mean ± SD. Different letters (a, b, c) in the same line indicate significant differences (P <0.05, Metastats)*.

### Correlation Analysis of Differential Microorganisms and Differential Metabolic Pathways

**Figure 6:**
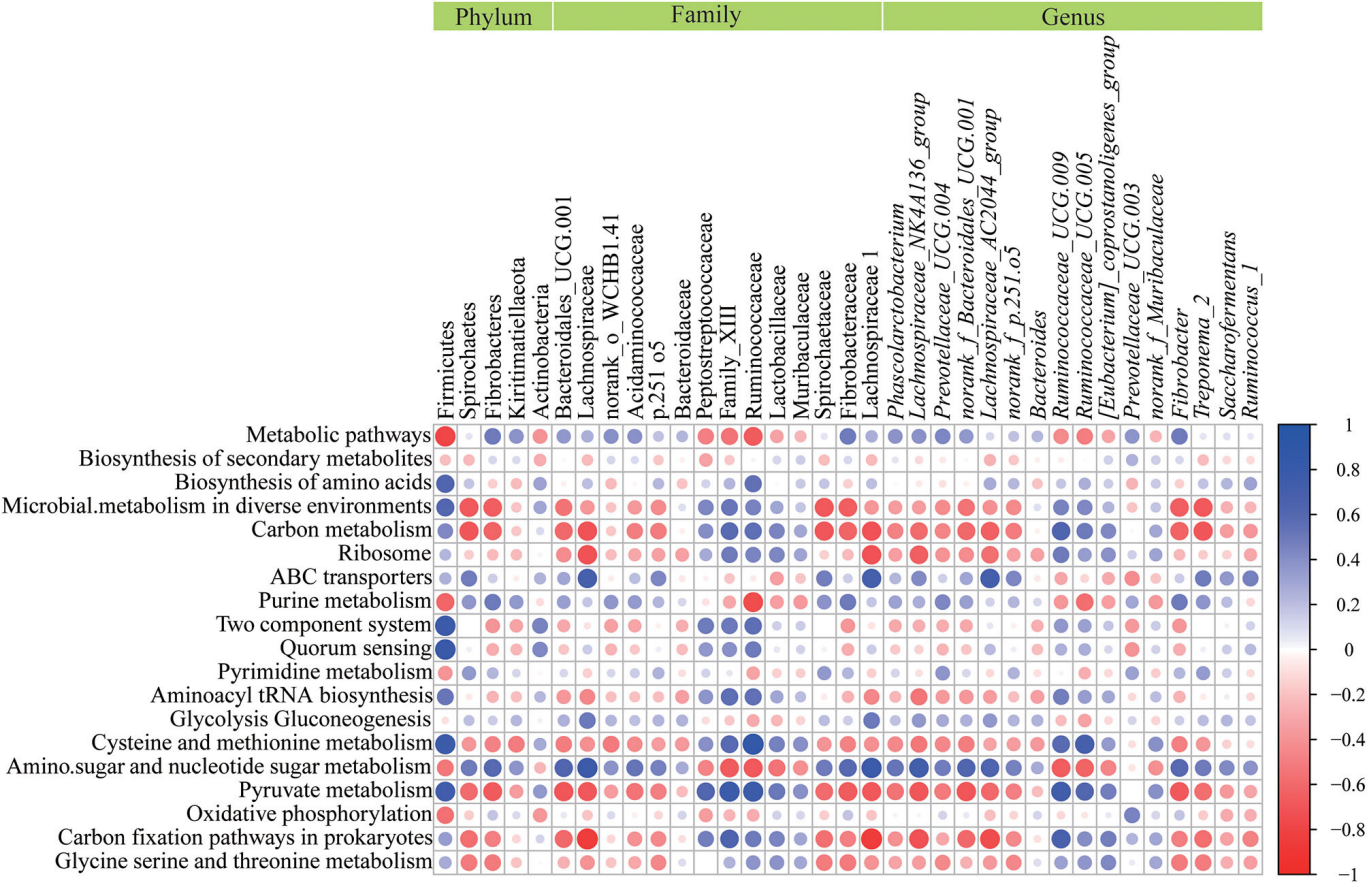
Correlation analysis between microorganisms and metabolic pathways affected by seasonal changes. In the graph, each line describes a metabolic pathway, and each column describes a phyla, family, or genus. Each circle represents the Pearson coefficient between a microorganism and a metabolic pathway. Blue represents positive correlation, while red represents negative correlation.

As shown in Figure 6, the results of the correlation analysis showed that Firmicutes positively correlated (*P* < 0.05) with cysteine and methionine metabolism, quorum sensing, pyruvate metabolism, biosynthesis of amino acid, two component system, aminoacyl tRNA biosynthesis, but was negatively correlated (*P* < 0.05) with oxidative phosphorylation, purine metabolism and metabolic pathways. Spirochaetes had a negative correlation (*P* < 0.05) with pyruvate metabolism and carbon metabolism. Lachnospiracea was positively correlated (*P* < 0.05) with amino sugar and nucleotide sugar metabolism, ABC transporters and glycolysis gluconeogenesis, but negatively (*P* < 0.05) correlated with carbon metabolism, pyruvate metabolism and carbon fixation pathways in prokaryotes. Peptostreptococcaceae had a positive correlation (*P* < 0.05) with pyruvate metabolism. Ruminococcaceae was positively correlated (*P* < 0.05) with pyruvate metabolism, carbon fixation pathways in prokaryotes, carbon metabolism, cysteine and methionine metabolism, biosynthesis of amino acids and aminoacyl tRNA biosynthesis, and negatively correlated (*P* < 0.05) with purine metabolism, metabolic pathways and amino sugar and nucleotide sugar metabolisms. Spirochaetaceae had a negative correlation (*P* < 0.05) with pyruvate metabolism and carbon metabolism. Lachnospiraceae was positively correlated (*P* < 0.05) with amino sugar and nucleotide sugar metabolism, ABC transporters glycolysis, and gluconeogenesis, but had a negative correlation (*P* < 0.05) with carbon metabolism, pyruvate metabolism and carbon fixation pathways in prokaryotes. *Ruminococcaceae_UCG 009* had a positive correlation (*P* < 0.05) with pyruvate metabolism, carbon fixation pathways in prokaryotes, carbon metabolism, and cysteine and methionine metabolism, and a negative correlation (*P* < 0.05) with amino sugar and nucleotide sugar metabolism. *Ruminococcus _UCG 005* was positively correlated (*P* < 0.05) with the metabolism of cysteine, methionine and pyruvate, and negatively correlated (*P* < 0.05) with the metabolism of purines, amino acids and nucleotide sugars. Pyruvate metabolism had a positive correlation (*P* < 0.05) with *[Eubacterium]_coprostanoligenes_group* and a negative correlation (*P* < 0.05) with Fibrobacter and *Treponema_2*. *Prevotellaceae_UCG 003* had a positive correlation (*P* < 0.05) with oxidative phosphorylation and metabolic pathways.

### Blood Physiological Parameters

Seasonal changes had a significant impact on blood physiological indicators (Table 3). In spring, WBC, Neu, Bas, Neu% and MPV were significantly higher than the other groups (*P* < 0.05); Lymph% was significantly lower than in other seasons. In summer, Eos%, MCHC, and RDW-CV were significantly higher than other seasons, whereas Bas, Neu%, MCV were observed significantly lower (*P* < 0.05). In autumn, RDW-SD, PLT and PCT were significantly lower than other seasons (*P* < 0.05). Lymph% and Mon% in winter were significantly higher than other seasons, whereas Neu% was significantly lower than other seasons (*P* < 0.05).

**Table 3:** Effects of seasonal changes on serum physiological parameters.

**Items**	**Spring**	**Summer**	**Autumn**	**Winter**	***P*-value**	**Reference range**
WBC, 10^9^/L	7.97 ± 2.78a	6.04 ± 1.79b	6.36 ± 1.38b	6.65 ± 1.25b	<0.001	5.10–15.80
Neu, 10^9^/L	3.71 ± 2.16a	2.09 ± 0.95bc	2.46 ± 1.00b	1.91 ± 0.58b	<0.001	1.32–8.96
Lymph, 10^9^/L	3.57 ± 1.01ab	3.39 ± 1.13b	3.37 ± 1.15b	3.98 ± 0.98a	0.019	2.01–7.80
Mon, 10^9^/L	0.45 ± 0.28ab	0.33 ± 0.19c	0.35 ± 0.31bc	0.54 ± 0.30a	<0.001	0.00–1.52
Eos, 10^9^/L	0.20 ± 0.27	0.21 ± 0.12	0.14 ± 0.08	0.19 ± 0.08	0.149	0.00–1.08
Bas, 10^9^/L	0.04 ± 0.02a	0.02 ± 0.02c	0.03 ± 0.02b	0.03 ± 0.01b	<0.001	0.00–0.17
Neu%, %	43.77 ± 12.09a	34.18 ± 8.69c	38.48 ± 12.11b	28.82 ± 6.98d	<0.001	21.50–68.00
Lymph%, %	47.27 ± 11.45c	56.37 ± 8.99ab	53.13 ± 12.97b	59.83 ± 8.88a	<0.001	28.00–64.50
Mon%, %	5.83 ± 3.59b	5.50 ± 2.23b	5.58 ± 4.95b	8.04 ± 3.89a	0.002	0.00–14.30
Eos%, %	2.56 ± 2.78b	3.52 ± 1.48a	2.27 ± 1.11b	2.79 ± 1.02b	0.004	0.00–8.00
Bas%, %	0.56 ± 0.24a	0.43 ± 0.27b	0.54 ± 0.25a	0.53 ± 0.22ab	0.041	0.00–1.50
RBC, 10^12^·L	9.01 ± 1.09	8.78 ± 0.95	8.75 ± 1.13	9.10 ± 1.10	0.296	5.50–14.20
HGC g/L	103.10 ± 11.68a	98.08 ± 11.10b	97.04 ± 11.02b	102.68 ± 11.22a	0.012	6–132
HCT, %	33.90 ± 3.78ab	31.32 ± 3.71c	32.37 ± 3.99bc	34.68 ± 3.99a	<0.001	20.00–39.00
MCV, fL	37.79 ± 2.75a	35.66 ± 1.84b	37.09 ± 2.55a	38.04 ± 2.32a	<0.001	25.00–41.00
MCH, pg	11.48 ± 0.68a	11.17 ± 0.59b	11.14 ± 0.66b	11.35 ± 0.61ab	0.031	8.00–12.30
MCHC, g/L	304.26 ± 13.15b	313.50 ± 13.48a	300.62 ± 13.79bc	296.29 ± 13.53c	<0.001	290–360
RDW-CV, %	19.56 ± 1.25bc	20.62 ± 2.07a	19.12 ± 1.06c	19.82 ± 1.67b	<0.001	16.50–26.20
RDW-SD, fL	30.54 ± 3.12a	31.27 ± 3.52a	28.86 ± 2.65b	30.38 ± 2.87a	0.001	20.00–35.00
PLT, 10^9^·L	308.56 ± 170.77a	310.9 ± 146.63a	147.74 ± 115.37c	214.12 ± 127.20b	<0.001	100–800
MPV, fL	5.83 ± 0.60a	5.54 ± 0.40b	5.02 ± 0.58c	5.49 ± 0.49b	<0.001	3.50–6.00
PDW, %	15.6 ± 0.44	15.43 ± 0.33	15.4 ± 0.47	15.52 ± 0.33	0.062	12.00–17.50
PCT, %	0.18 ± 0.10a	0.17 ± 0.08a	0.08 ± 0.06c	0.12 ± 0.08b	<0.001	0.05–0.42

*The value shown are the mean ± SD. In the same line, different letters (a, b, c, d) represent significant differences (P <0.05). Neu, neutrophils; Bas, basophils; HCT, haematocrit; WBC, the leukocytes; Lymph, lymphocytes; MCHC, mean corpuscular hemoglobin concentration; Mon, monocytes; Eos, eosinophils; Neu%, percentage of neutrophils; Lymph%, percentage of lymphocytes; Mon%, percentage of monocytes; Eos%, percentage of eosinophils; Bas%, percentage of basophils; RBC, erythrocytes; HGB, hemoglobin; MCH, mean corpuscular hemoglobin; RDW-CV, coefficient of variation of red blood cell distribution width; RDW-SD, standard deviation of red blood cell distribution width; PLT, platelet count; MCV, mean corpuscular volume; MPV, mean platelet volume; PDW, platelet distribution width; PCT, platelet crit*.

## Discussion

The gut microbiome plays a key role in the overall life of animal and has important implications on the physiology and metabolism of animals. Factors such as host's body size, physical condition, and seasonal changes in diet also affect the structural composition of the gut microbial community (21–23). The blood physiological atlas provides a reliable information about the health of animals, and also reflects their response to internal and external environment (24, 25). However, as the season changes, intestinal microbes and blood physiology also change (26, 27). However, with seasonal changes, the relationship between intestinal microbes and blood physiological indicators are still unclear. Therefore, we determined the microbial community structure of rectal feces by high-throughput sequencing to provide reference for seasonal changes, relationships between gut microbes, and physiological blood indicators.

### Seasonal Effects on Intestinal Microbial Community Diversity and Structure

Affected by the combined effects of environmental factors (temperature, humidity, etc.), animals need to adapt to different climatic conditions, and small ruminants are one of the animals with the worst response to heat stress (28, 29). The result showed that significant differences in microbial diversity and abundance in different seasons, where the summer had the highest microbial diversity and abundance. Under the environmental conditions of high temperature and humidity, the fluidity of rumen of ruminants was inhibited, which increased the residence time of feed in rumen and improved the utilization rate of feed dry matter, resulting in the improvement of microbial diversity and richness in summer (30–32).

With seasonal changes, the main phyla of intestinal microbes in Chinese merino fine-wool sheep did not change, which mainly included Firmicutes and Bacteroidetes. Previous studies also reported that the dominant phylum in ruminant fecal samples did not change in different seasons (33, 34). According to the LEfSE analysis, Kiritimatiellaeota was the phylum with relatively high abundance in the spring. Kiritimatiellaeota belongs to the planktonic bacteria-Verrucobacterium-Chlamydia (PVC) superphylum, and its main function is to degrade complex polysaccharides and glycoproteins to obtain energy (35, 36). Firmicutes were the representative mycophyta in autumn. Firmicutes can help the host degrade fiber and cellulose, and contribute to energy intake and nutrient absorption. In the cold winter, Spirochaetes, Fibrobacteres and Actinobacteria were the representative bacteria phyla. Fibrobacteres was considered to be the main degrading bacteria of lignocellulose in the intestines of herbivores (37). Spirochaetes was also an important lignocellulose degrading bacteria (38). It is worth noticing that although Actinobacteria is relatively small, however it is very important in maintaining the homeostasis of the sheep's intestinal tract. In particular, Bifidobacterium is a widely used probiotic food supplement (39).

At family level, consistent with the study of Xia et al. (40), Ruminococcaceae was the dominant microbiota in the intestine and is not affected by the season. Ruminococcaceae is the main microorganisms in the intestinal tract of ruminants and is important for the degradation of cellulose and starch (21, 41, 42). In addition, we found that Lachnospiraceae were relatively enriched in cold seasons (Spring and Winter), and Lachnospiraceae may be used as a marker of cold stress. When mice were fed cold nature herb, *Lachnospiraceae uncultured* dominated the large intestine (43). Lachnospiriaceae were more common in the cecum of Brucella rats under cold-induced conditions (44). Research showed that Lachnospiriaceae was involved in the digestion and absorption of polysaccharides, producing lactic acid and increasing butyrate levels in feces (45, 46). It was believed to improve the intestines of omnivores. The increase of Lachnospiraceae in the cold season improved the digestion of polysaccharides and produced more energy.

At genus level, *Ruminococcaceae_UCG-005, Rikenellaceae_RC9_gut_group, Christensenellaceae_R-7_group, Ruminococcaceae_UCG-010* were the most important microorganisms in the four seasons. Both *Ruminococcaceae_UCG-005* and *Ruminococcaceae_UCG-010* belong to Ruminococcaceae, and *Christensenellaceae_R-7_group* belongs to Christensenellaceae. Ruminococcaceae and Christensenbacteriaceae are considered to be potentially beneficial bacteria that can regulate the internal environment of the intestine and are related to immune regulation and healthy homeostasis (47, 48). *Rikenellaceae_RC9_gut_group*, in intestine of rats with acute necrotizing pancreatitis, and dairy cows receiving oil supplements was significantly reduced according to previous reports (49, 50). But the specific function of this group was still not very clear. In addition, it has been reported that the increase of Rikenellaceae was associated with a healthy metabolic state (51). *Phascolarctobacterium* was more enriched in the spring. It can colonize the human gastrointestinal tract and makes short-chain fatty acids (SCFA) (acetic acid and propionic acid). SCFA and their receptors play important roles in physiology and pathology of the gastrointestinal tract, and are involved in host metabolism and mood regulation (52). *Bacteroides*, a kind of beneficial bacteria in the intestine, were more abundant in summer. These can use carbohydrates to stimulate the production of fucosylated glycans in the inner wall of the intestine, which can improve the metabolism and immune dysfunction of induced obese mice, and support the formation of neonatal blood vessels (53–55). The bacteria enriched in autumn included *Ruminococcaceae_UCG-005, Ruminococcaceae_UCG-009*, and *Prevotellaceae_UCG-003*, all of which have the function of carbohydrates degradation (41, 56). This might be because the autumn season were more suitable, and the Chinese merino fine-wool sheep improved the utilization of carbohydrates and carried out their own fattening. *Fibrobacter, Treponema_2, Saccharofermentans* and *Ruminococcus_1* were higher in winter. Fan's et al. (57) results showed that *Ruminococcus_1* was more concentrated in summer, while our results showed that *Ruminococcus_1* and *Saccharofermentans* were more concentrated in winter, which may be due to relatively stable source of nutrients. In addition, in the cold winter, more energy was needed to resist the cold.

### Seasonal Effects on Intestinal Microbial Metabolic Pathways

The gut microbiome is critical to the host, especially with regard to host health, metabolism, digestion, and immunity (47, 58, 59). Intestinal microorganisms carry a large number of genes. These genes can help the host regulate composition of microbes in the microbial population, thereby helping the host to adapt the complex external environment (60–62). In this research, PICRUSt was used to forecast gut microbial function of Chinese merino fine-wool sheep. In addition, with the change of seasons, the host's KEGG metabolic pathways also undergo certain changes (63, 64). In this experiment, the Chinese merino fine-wool sheep pathway predicted by KEGG (level 3) was also significantly different.

The results showed that the metabolic pathway of ABC transporters in autumn was significantly decreased than that in other seasons, and there was a significant positive correlation with Lachnospiraceae. Ma et al. (65) confirmed that the metabolic function of ABC transporters in yak increased significantly in severe weather. Compared with other seasons in this experiment, autumn was in the most suitable environmental climate. ABC transporters consist of two ABC or nucleotide-binding domains with highly conserved sequence motifs and two transmembrane domains, which are a protective barrier to protect the body from toxic substances (66, 67). In addition, researchers have reported that ABC transporters were involved in the underlying mechanism of inflammation (68). Lachnospiraceae can use fiber in feed to produce SCFA (butyric acid), and butyric acid can be used as a substrate for the synthesis of ABC transporters (46, 69). Moreover, pyruvate metabolism in autumn was significantly increased than that in other seasons, and it was a strong positive correlation with Peptostreptococcaceae and Ruminococcaceae. Pyruvate is the final product of glycolysis and plays a very important role in cell metabolism. Under anaerobic conditions, pyruvate will produce lactic acid under the action of lactate dehydrogenase. In the aerobic state, pyruvate enters the mitochondria, oxidative decarboxylation produces acetyl-CoA, which increases the flux into the tricarboxylic acid cycle (70). In anaerobic or aerobic state, the decomposition of pyruvate can provide energy for physiological activities of livestock and poultry. Therefore, in this experiment, Chinese merino fine-wool sheep might enhance pyruvate metabolism in autumn with good air environment quality, which was beneficial to its own growth and development. Peptostreptococcaceae is an anaerobic bacterium that can decompose D, L-lactic acid to produce propionic acid, butyric acid and isovaleric acid (71). Ruminococcaceae can degrade the fiber in the feed to produce SCFAs (mainly butyric acid) (72, 73). In the process of producing acetic acid and butyrate, Ruminococcaceae needs to first produce acetyl-CoA, whereas pyruvate metabolism is the main source of acetyl-CoA (74).

### Mechanism of Digestive Tract Microorganisms Affecting Blood Physiology in Seasons

Physiological indicators of blood are very important for farms to assess the physiological conditions of livestock and poultry (75). Seasonal change can directly affect the physiological function of animal, and its physiological indexes will change accordingly (19, 76). Studies have reported that with the change of seasons, sheep's blood physiological indicators will also change (77).

The results showed that with the seasonal changes, all the physiological indicators of Chinese merino fine-wool sheep were within a reasonable range and in a healthy state. In addition, the number of WBC, Neu and basophils in the spring were significantly higher than those in other seasons. Jia et al. (78) reported that in mice PM_2.5_ could significantly increase the number of Leu, Neu, macrophages, Lymph and Eos which was in line with our findings. There was a certain degree of consistency in our results. The main functions of WBC and their secretions are to fight against infection, protect the body from invasion by foreign organisms through phagocytosis, and produce or at least transport and distribute antibodies in the immune response (75). Increased WBC counts may indicate an increase in the likelihood of infection (79). In addition, previous studies showed that Neu in mice exposed to PM_2.5_ for a long time was increased, and cold stress (0°C) can also enhance its effect (80). In addition, PM_2.5_ would also synergistically activate the activity of basophils and significantly increase the specific antigen IgE in mice (81). Dai's et al. (82) results showed that overexpression of ABC transporter-A1 could reduce ovalbumin-induced airway neutropenia, IgE and airway epithelial remodeling. Therefore, the pyruvate metabolism of fine-wool sheep was enhanced in autumn when the environmental climate was better, which may be due to less inflammatory damage and the down-regulation of ABC transporters metabolic pathway.

Hot and cold stress is a huge challenge for livestock and poultry (especially young animals) to survive. Our study found that in the three seasons of spring, summer and winter when cold and heat stress was more obvious, the metabolic pathway of ABC transporter was significantly increased than that in autumn. In addition, it was also found that Lachnospiraceae and its genus-level microorganisms were enriched in spring, summer and winter seasons, and had a strong correlation with ABC transporters. We speculate that under the influence of cold and heat stress, Lachnospiraceae was relatively abundant. The increase in the degree of Neu leads to changes in the metabolic pathways of ABC transporters, which in turn affect the changes in Neu, but further studies are required for the confirmation.

## Conclusion

In summary, seasonal changes affected the microbial community structure and blood physiological indicators of the intestinal microbes. Compared with other seasons, summer had the highest diversity. In different seasons, the dominant intestinal microbes had not changed. The results of LEfSe analysis showed that Lachnospiraceae had relatively high abundance in spring; in summer, the relatively abundant flora family was Bacteroidaceae; in autumn, Ruminococcaceae was more abundant; whereas Spirochaetaceae and Fibrobacteraceae were relatively abundant in winter. PICRUSt function prediction results showed that metabolic pathways changes in different seasons. ABC transporters pathway in autumn was significantly decreased than other seasons, which indicted that with seasonal changes, gut microbes played an important role. The amount of WBC, Neu and Bas in spring was higher. In addition, from the results of correlation analysis, we could get that lachnospiraceae had a significant positive correlation with ABC transporters. This indicated that the changes in Lachnospiraceae lead to changes in ABC transporters metabolic pathways, which in turn affect the changes in Neu, but further studies are needed for confirmation. Therefore, in the cold season, farms can increase the level of butyric acid in young animals by regulating nutrient levels to induce the metabolic pathway of ABC transprotein, increase the metabolism of pyruvate, and enhance the resistance to harsh environments.

## Data Availability Statement

The datasets presented in this study can be found in online repositories. The names of the repository/repositories and accession number(s) can be found in the article/Supplementary Material.

## Ethics Statement

This study was carried out in accordance with the Guidelines for the Care and Use of Laboratory Animals of the Scientific Research Department of Xinjiang Academy of Agricultural and Reclamation Sciences (protocol approval number: XJNKKXY-AEP-038). This study did not involve any endangered or protected animal species. Individual oral/written informed consent for the use of samples was obtained from all the animal owners. Written informed consent was obtained from the owners for the participation of their animals in this study.

## Author Contributions

FZ, XZ, MW, and PZ designed the experiments and reviewed the manuscript. Samples were collected at XZ, YG, MH, XH, LW, YZ, HT, and QZ. XZ and TW conducted experiments. XZ and CL analyzed the data and completed the manuscript. XZ, CL, MW, and KS prepared images and edited the manuscript. All authors reviewed the manuscript.

## Funding

This study was supported by the Ring-Fenced Funding of Major projects of State Key Laboratory for Sheep Genetic Improvement and Healthy Production (2021ZD05 and 2021ZD07); Outstanding young and middle-aged talents Training Project of State Key Laboratory for Sheep Genetic Improvement and Healthy Production (SKLSGIHP2017A03 and SKLSGIHP2021A03); and National Major Project of Transgenic Biotechnology Breeding (2016ZX08008001).

## Conflict of Interest

The authors declare that the research was conducted in the absence of any commercial or financial relationships that could be construed as a potential conflict of interest.

## Publisher's Note

All claims expressed in this article are solely those of the authors and do not necessarily represent those of their affiliated organizations, or those of the publisher, the editors and the reviewers. Any product that may be evaluated in this article, or claim that may be made by its manufacturer, is not guaranteed or endorsed by the publisher.
