# Supplementary material for: Seasonal Differences in Fecal Microbial Community Structure and Metabolism of House-Feeding Chinese Merino Fine-Wool Sheep
Source: Front Vet Sci. 2022 Mar 23;9:875729. doi: 10.3389/fvets.2022.875729 (PMC8989412; doi:10.3389/fvets.2022.875729)
Supplement: Supplementary file 1 [file Data_Sheet_1.docx]

**Table S1**: Ingredients and nutrient composition of experimental diets offered to house-feeding Chinese merino fine-wool sheep

| Items | Content |
| --- | --- |
| Ingredients (% of DM) | |
| Corn | 10.00 |
| Cotton seed meal | 5.10 |
| Bone meal | 1.00 |
| Corn Silage | 26.40 |
| Tomato pomace | 5.00 |
| Alfalfa hay | 45.00 |
| Cottonseed Hull | 4.25 |
| Premix^1^ | 3.25 |
| Total | 100.00 |
| Nutrient composition ^2^ | |
| Metabolizable energy (ME) (MJ/Kg of DM) | 7.23 |
| Crude protein (CP) (% of DM) | 12.34 |
| Neutral detergent fiber (NDF) (% of DM) | 35.00 |
| Acid detergent fiber (ADF) (% of DM) | 17.46 |
| Ca (% of DM) | 0.76 |
| P (% of DM) | 0.44 |

Note: ^1^ The premix provided the following per kg of diets: Vitamin A 300 IU, Vitamin D_3_ 420 IU, Vitamin E 5 IU, biotin 0.16 mg, pantothenic acid 4.2 mg, nicotinic acid 3.6 mg, Cu: 2 mg, Fe: 22 mg, Mn: 20 mg, Zn: 16 mg, I: 0.4 mg, Se: 0.13 mg, Co: 0.13 mg;

^2^ Nutrient composition were measured values except for Metabolizable energy.

**Table S2:** Results of bray_curtis PERMANOVA analysis

| Item | SumsOfSqs | MeanSqs | F.Model | R^2^ | P.value | P.adjust |
| --- | --- | --- | --- | --- | --- | --- |
| Seasons | 4.277 | 1.426 | 12.531 | 0.161 | 0.001 | 0.001 |

Note: The R^2^ value represents the explanation degree of the grouping factor to the sample difference. The larger the R2 is, the higher the explanation degree of the grouping is for the difference; the P value less than 0.05 indicates that the reliability of this test is high.

**Table S3:** PERMANOVA analysis between the seasonal comparisons.

| Season | Statistic | *P*-value | Permutation_number |
| --- | --- | --- | --- |
| Spring-Summer | 0.013 | 0.115 | 999 |
| Spring-Autumn | 0.679 | 0.001 | 999 |
| Spring-Winter | 0.091 | 0.001 | 999 |
| Summer-Autumn | 0.747 | 0.001 | 999 |
| Summer-Winter | 0.080 | 0.001 | 999 |
| Autumn-Winter | 0.740 | 0.001 | 999 |
